# The Impact of Various Platelet Indices as Prognostic Markers of Septic Shock

**DOI:** 10.1371/journal.pone.0103761

**Published:** 2014-08-13

**Authors:** Yanxia Gao, Yi Li, Xuezhong Yu, Shigong Guo, Xu Ji, Tongwen Sun, Chao Lan, Valery Lavergne, Marc Ghannoum, Li Li

**Affiliations:** 1 Emergency Department, The First Affiliated Hospital of Zhengzhou University, Zhengzhou, P.R.China; 2 Emergency Department, Peking Union Medical College Hospital, Beijing, P.R.China; 3 St Mary's Hospital, London, United Kingdom; 4 Clinical Specimen Bank, Chinese PLA General Hospital, Beijing, China; 5 Integrated Intensive Care unit, The First Affiliated Hospital of Zhengzhou University, Zhengzhou, P.R.China; 6 Department of Medical Biology, Sacré-Coeur Hospital, University of Montreal, Montreal, Québec, Canada; 7 Department of Specialized Medicine, Verdun Hospital, University of Montreal, Montreal, Québec, Canada; University of Leicester, United Kingdom

## Abstract

**Introduction:**

Platelet indices, including mean platelet volume (MPV), are readily available blood tests, although their prognostic value in patients with septic shock has not been fully explored. Current evidence has found contradictory results. This study aims to explore the behavior of platelet indices in septic shock and their clinical prognostic value.

**Methods:**

Charts of septic shock patients from January to December 2012 in a tertiary medical center in Northern China were reviewed retrospectively. Platelet indices were recorded during the first five consecutive days after admission, as well as the penultimate and the last day of hospital stay. The data were compared between surviving and non-surviving patients.

**Results:**

A total of 124 septic shock patients were enrolled. Thirty-six of the patients survived and 88 of them expired. MPV in the non-survivor group was higher than that of the survivor group, especially on the last day. PDW and PLCR showed increased trends, while PCT and PLT decreased in the non-survivor group. Among the PLT indices, MPV had the highest area under the receiver operating characteristic curve (0.81) with a precision rate of 75.6% at a cut-off of 10.5.Compared with other more usual septic shock prognostic markers, MPV is second only to lactate for the highest area under the curve.

**Conclusion:**

A statistically significant difference was seen between survivors and non-survivors for platelet indices which make them easily available and useful prognostic markers for patients in septic shock.

## Introduction

Septic shock is a major healthcare problem, affecting millions of people worldwide and carries a 25% mortality rate [1]. Changes in the coagulation system is involved in septic shock, which is manifested by the prolongation of the activated partial thromboplastin time (aPTT) and prothombin time (PT), and decreased platelet (PLT) count [2], [3]. The extent of the PLT fall is correlated to the prognosis, and PLT returns towards normal values as the patient recovers [4], [5].

Mean platelet volume (MPV) is a platelet index that has been available since the 1970s [6]. Since then, other indices of platelets have been introduced, including platelet volume distribution width (PDW), plateletcrit (PCT), and platelet large cell ratio (PLCR). All these indices can be measured by an inexpensive and readily available routine blood count; however their use and application in septic shock remains unknown [7].

Of the four major platelet indices, MPV changes have been already observed in some infected patients, such as those presenting with acute appendicitis [8], pancreatitis [9], infective endocarditis [10], and malaria [11], although the evidence in septic shock is currently controversial: some studies suggest that MPV increases during septic shock [12], [13], whereas others conversely show a decrease [14]. Van der Lelie et al found that half of patients diagnosed with sepsis had an increased MPV, and suggested that an increased MPV could be associated with invasive infections [12]. Similarly in a canine model of endotoxemia, MPV and PDW were observed to increase while PLT and PCT decreased in septic subjects compared to controls, which prompted the authors to suggest using platelet indices in the diagnosis and monitoring of endotoxemia. [13]. On the other hand, Bessman et al found that MPV decreased during sepsis; they studied 9 septic patients and found that they had decreased MPV and platelet count. Six of these patients recovered with MPV normalizing more rapidly to its normal range than the PLT count [14].

The PDW increases during platelet depletion when turnover is accelerated, and shares similar behavior to MPV during acute severe infections. PLCR is another surrogate marker for the platelet volume, which identifies the largest-sized fraction of platelets. An increase in PLCR usually signifies that there is an increase in new platelets (which are larger in size). PCT is the plateletcrit and is influenced by the number and the size of platelets, and has a positive relationship with the platelet count.

In view of the above mentioned discrepancies, we analyzed changes in MPV and other platelet indices in septic shock patients and compared them between survivors and non-survivors. They were also compared to other septic indices, such as lactate and procalcitonin and other indices from a routine blood count, such as the white blood cell (WBC) count and the hematocrit (HCT). Since platelets are of a larger size when newly produced from the bone marrow and then subsequently decrease in size, we hypothesize that MPV has a greater impact in non-survivors and is positively correlated with disease severity. Our objectives were to identify if MPV and other platelet indices have an impact on the prognosis of septic shock.

## Materials and Methods

### Patients and study design

This retrospective cohort study was carried out in a tertiary medical center of over 1800 beds disserving a large area in the north of China.

From January to December 2012, all patients diagnosed with septic shock were evaluated for inclusion in the study. Exclusion criteria were as follows: patients with concomitant hematological diseases (hematological malignancies, autoimmune thrombocytopenic purpura, reactive thrombocytosis, and hypersplenism), patients receiving platelets or fresh frozen plasma, and pregnant or breastfeeding women. All medical charts were independently reviewed by two physicians: if there was discrepancy between them regarding patient inclusion, all other authors were consulted to reach agreement.

The complete blood counts (CBCs) performed on the first five days after admission, on the penultimate and last day of hospital stay were reviewed. The CBC included: the PLT count, MPV, PDW, PLCR, PCT, HCT and WBC count. The CBCs were performed using a Sysmex XE 5000 analyzer (Japan).The normal range of PLT, MPV, PDW, PLCR, and PCT were 100–300×10^9^/L, 7–13 fl, 9–17%, 13–43%, and 0.11–0.28% respectively. Lactate, procalcitonin and calcium levels as well as hepatic and renal functions, performed on the same days as the CBC, were also reviewed.

### Definitions

Accordingly to Dellinger's criteria, septic shock was defined as “sepsis induced hypotension persisting despite adequate fluid resuscitation”, sepsis as “infection plus systemic manifestations of infection” and hypotension as “systolic blood pressure (SBP) of <90 mm Hg or mean arterial pressure <70 mm Hg or a SBP decrease >40 mm Hg or <2 SD below normal for the patient's age” [1].

### Ethical statement

After consulting the Ethics Department of the First Affiliated Hospital of Zhengzhou University, ethical approval was waived since our study was a non-interventional retrospective study involving anonymized patient data.

### Statistical analysis

The cohort was divided in 2 groups based on their final outcome (survivor and non-survivor groups). Groups were stratified according to the treatment received (surgical intervention, if performed or not). Continuous variables were expressed as means with standard deviations or medians with interquartile ranges, and categorical variables as numbers with percentages. In order to compare groups, either one-way ANOVA or non-parametric tests were used for continuous variables, accordingly to the homogeneity of variance test. In order to establish the predictive value of each studied parameters for mortality, receiver operating characteristic (ROC) curves were plotted for HCT, MPV, WBC, lactate, procalcitonin and APACHE II score. All p-values of less than 0.05 were considered statistically significant. Statistical analysis was conducted with SPSS 18.0 software (SPSS Inc., Chicago, IL, USA).

## Results

**Figure 1:**
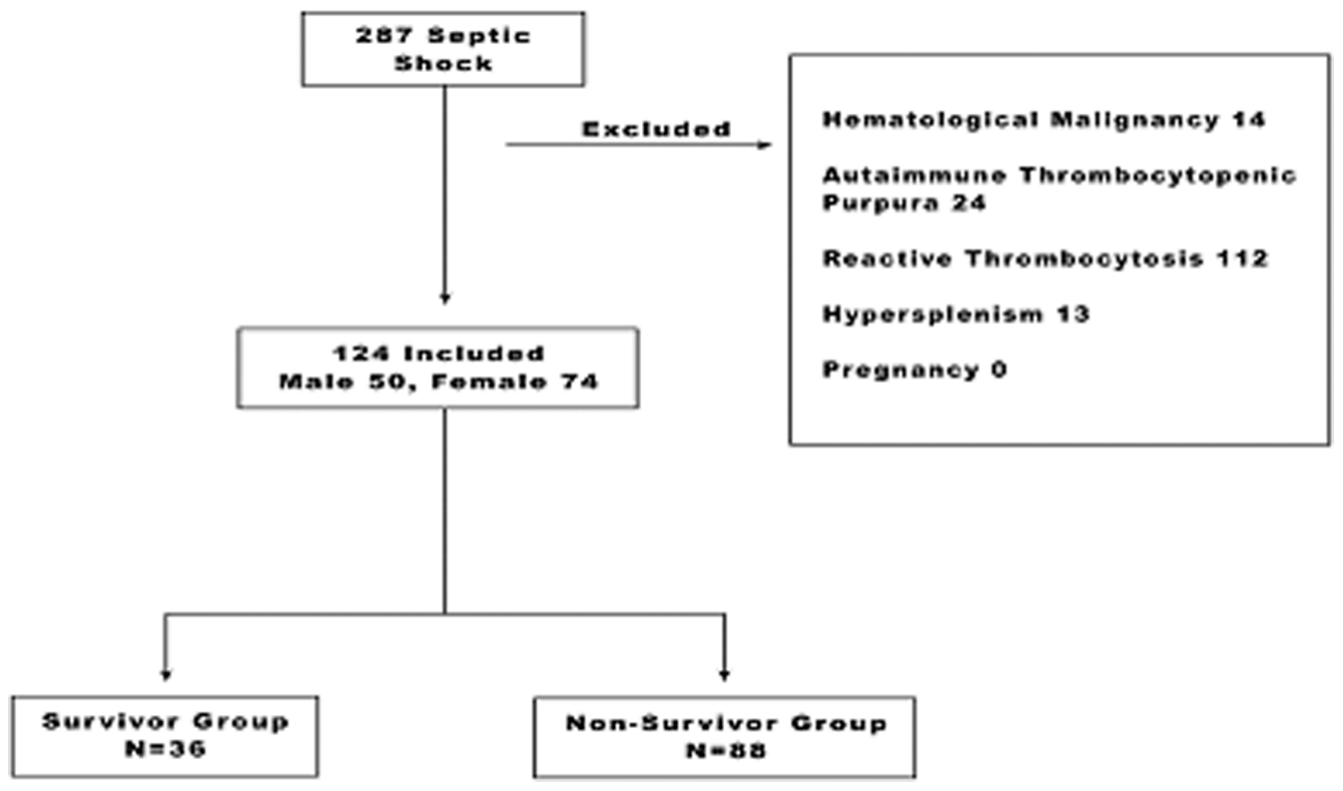
Flowchart of the enrollment of septic shock patients.

During the study period, a total of 287 patients with septic shock were evaluated from which 124 were included. Overall, 88 patients died during the course of their hospitalization, while 36 were discharged alive (figure 1). A total of 50 men and 74 women were studied. The characteristics on admission are shown in table 1. When comparing survivors and non-survivors on admission, there were no differences in demographics, vital signs, microbiology data, infectious focus or surgical intervention but the APACHE II score was statistically higher in the non-survivor group (p = 0.04). A total of 4 patients underwent surgical intervention in the survivor group, while 10 patients did in the non-survivor group. The coagulation parameters (PT, aPTT and fibrinogen) were comparable on admission.

**Table 1:** Patients' characteristics on admission comparing survivors and non-survivors.

	Survivor group (n = 36)	Non-survivor group(n = 88)	P-value
Age	61.17±20.639	61.70±17.875	0.82
Temperature	37.62±1.19	37.59±1.071	0.52
Respiratory	23.03±8.453	24.59±6.933	0.17
Heart rate	105.44±25.897	108.76±30.65	0.83
White cell count	10.9761±10.533	11.0959±8.653	0.98
Microbiology	25	63	0.54
Gram negative rods	18	52	0.45
Gram positive cocci	5	8	0.23
Fungi	2	3	0.13
Infection acquired			
Community	24	63	0.32
Hospital	12	25	0.43
Infectious focus			
Pulmonary	16	56	0.23
Intra-abdominal	14	23	0.14
Urologic	3	3	0.21
CNS	2	4	0.42
others	1	2	0.34
Surgical intervention	4	10	1.00
PT	15.21±2.31	16.31±2.51	0.23
aPTT	34.71±11.21	36.27±10.54	0.15
fibrinogen	3.21±2.25	3.32±2.14	0.31
number of patients with organ dysfuntion			
>3	7	13	0.48
2	24	64	0.32
1	5	11	0.45
number of patients on ventilation	21	69	0.54
APACHE II	30(28, 33)	35(27, 37.5)	0.04

**Figure 2:**
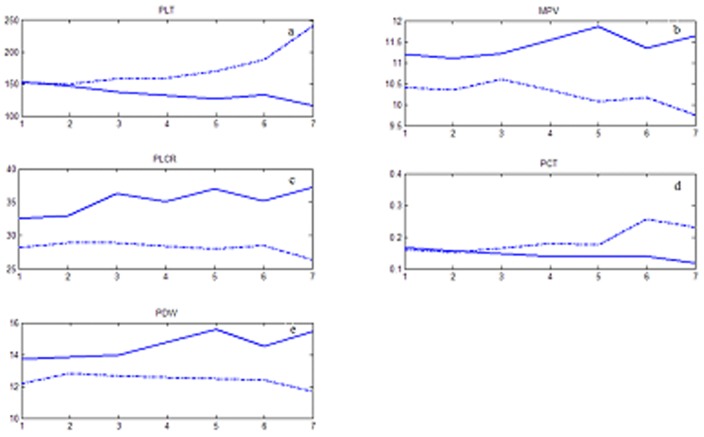
Change of platelet indices between groups.

On admission, all platelet indices were comparable between survivor and non-survivor groups, except for MPV which was statistically higher in the non-survivor group (p = 0.01). After stratification for surgical intervention, the same effect was only observed in the subgroup of patients in whom a surgical intervention was not performed (p = 0.03) (as shown in table 2). The longitudinal changes of each platelet index are shown on figure 2. In the non-survivor group, PLT counts and PCT tended to decrease with time, while MPV, PDW and PLCR increased.

**Table 2:** Comparison of platelet indices between survivors and non-survivors.

	Non-survivor group	Survivor group	P1 value	P2 value	P3 value
PDW	13.7 (11.90, 16.80)	11.7 (10.90, 13.43)	0.80	0.62	0.84
PLCR	33.65 (28.1, 42.75)	26.8 (23.2, 32.03)	0.32	0.20	0.40
PCT	0.12 (0.06, 0.20)	0.18 (0.09, 0.23)	0.75	0.60	0.85
PLT	105 (47.5, 191.5)	164 (85, 236)	0.44	0.40	0.59
MPV	11.2 (10.5, 12.5)	10.3 (9.68, 11)	0.01	0.03	0.84

All data are reported with medians (interquartile ranges). P1 values compare the total survivor group with the non-survivor group, P2 value compares the two groups after excluding patients who underwent surgical intervention, and P3 value compare the two group within those who underwent surgery.

**Figure 3:**
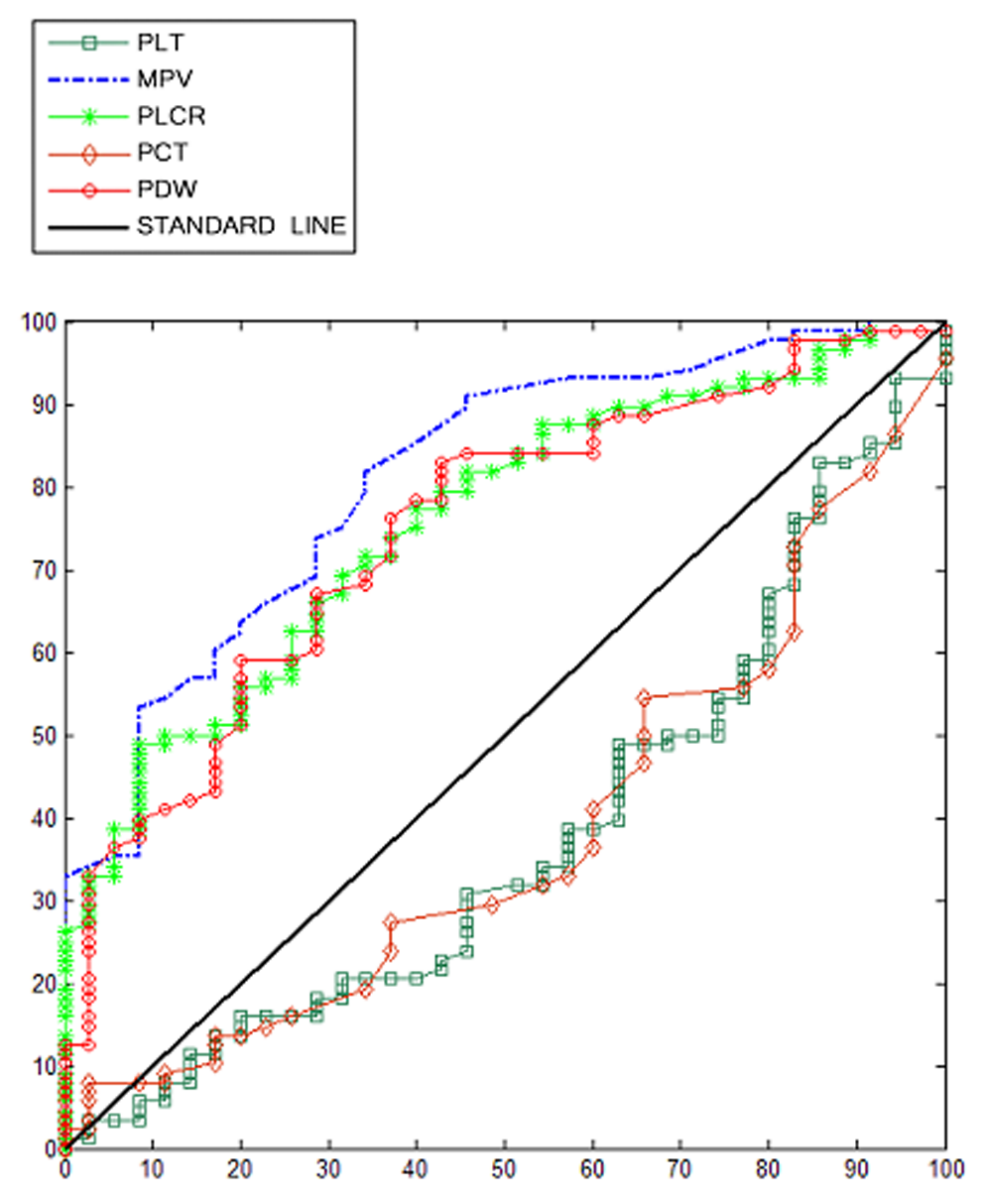
MPV, lactate, HCT, WBC, APACHE II and procalcitonin ROC curve map.

ROC curves were plotted daily for every platelet indices. All pertinent data are shown in table 3. MPV was the platelet indice showing the highest area under the curve (AUC of 0.81), with a sensitivity of 81.81% and a specificity of 65.71% at a cut-off of 10.5. Furthermore, MPV on the last day had the highest area under the curve (AUC 0.88, with a sensitivity of 81.82% and a specificity 85.71% of at a cut-off of 10.5) compared with the other days. PLCR and PDW showed the second and third highest AUC, (both are 0.76) while the PCT and PLT count showed the lowest AUC (both are 0.38.). As shown in figure 3, the MPV ROC curve was plotted with other parameters on admission. Lactate had the highest AUC (0.91), while MPV was the second best predictor of mortality. HCT, WBC, APACHE II and procalcitonin had lower AUC.

**Table 3:** ROC curve analysis for platelet indices at this different times.

	best cutoff (>)	Sensitivity (%)	Specifity (%)	accurate rate (%)	Youden index	AUC
PLT	487	1.13	100	29.27	0.01	0.38
PLT1	241	19.32	91.43	39.02	0.1	0.48
PLT2	279	18.18	91.43	38.21	0.1	0.46
PLT3	260	100	5.71	72.36	0.06	0.43
PLT4	245	15.91	88.57	35.77	0.04	0.42
PLT5	487	1.14	100	29.27	0.01	0.38
PLTLast2	476	1.14	100	29.27	0.01	0.35
PLTLast1	638	0	100	28.46	0	0.24
MPV	10.5	81.81	65.71	75.61	0.48	0.81
MPV1	10.9	59.09	77.14	63.41	0.36	0.69
MPV2	10.8	52.27	77.14	56.91	0.29	0.66
MPV3	10.7	67.05	65.71	65.04	0.33	0.66
MPV4	10	90.91	48.57	76.42	0.39	0.75
MPV5	10.5	81.82	65.71	75.61	0.48	0.81
MPVLast2	10	86.36	48.57	74.8	0.35	0.73
MPVLast1	10.5	81.82	85.71	81.3	0.68	0.88
PLCR	39.30	48.86	91.43	60.98	0.40	0.76
P_LCR1	32.1	51.14	80	58.54	0.31	0.63
P_LCR2	31.8	52.27	74.29	58.54	0.27	0.64
P_LCR3	29.3	70.45	68.57	69.11	0.39	0.69
P_LCR4	32	59.09	77.14	64.23	0.36	0.71
P_LCR5	39.3	48.86	91.43	60.98	0.4	0.76
P_LCRLast2	27.1	81.82	51.43	72.36	0.33	0.71
P_LCRLast1	27.7	85.23	74.29	81.3	0.6	0.82
PCT	0.33	7.95	97.14	32.52	0.05	0.38
PCT1	0.26	18.18	91.43	38.21	0.1	0.5
PCT2	0.36	7.95	100	33.33	0.08	0.49
PCT3	0.32	6.82	97.14	32.52	0.04	0.43
PCT4	0.27	13.64	88.57	34.15	0.02	0.42
PCT5	0.33	7.95	97.14	32.52	0.05	0.38
PCTLast2	2.2	0	100	28.46	0	0.35
PCTLast1	0.75	0	100	28.46	0	0.25
PDW	11.80	82.95	57.14	74.80	0.40	0.75
PDW1	13.4	52.27	82.86	60.98	0.35	0.66
PDW2	13	57.95	68.57	60.16	0.27	0.61
PDW3	11.7	75	54.29	67.48	0.29	0.64
PDW4	13.8	52.27	82.86	60.98	0.35	0.7
PDW5	11.8	82.95	57.14	74.8	0.4	0.75
PDWLast2	12.6	67.05	65.71	66.67	0.33	0.69
PDWLast1	12.3	71.59	80	72.38	0.52	0.81

PLT means the value of the mean value. PLT1, PLT2,PLT3, PLT4, PLT5 represent the values of day 1, day 2, day 3, day 4 and day 5 respectively. PLTlast2 and PLTlast1 represent the values of the penultimate and last day of hospital stay. The other platelet indices go as the same way.

## Discussion

In this retrospective study of 124 patients with septic shock, there were several important findings. First, in the non-survivor group, we observed that MPV, PLCR and PDW were increasing, while PLT and PCT were decreasing with time. Also, in the subgroup of patient not undergoing surgery, MPV on admission was higher in non-survivors compared to survivors. Most importantly, MPV over 10.5 on admission and on the first three days after admission was a good predictor of mortality in patients with septic shock.

The PLT count was lower in non-survivors, a finding that has already been described previously [2], [3]. This drop presumably arises from depletion of coagulation factors and platelet consumption during the septic process, and is a significant prognostic indicator of mortality.

The MPV is a useful test for the differential diagnosis of thrombocytopenia [21]. An increase in MPV, a sign of larger PLT size, usually is indicative of compensated bone marrow PLT production following stress-induced platelet destruction, as septic shock develops [12]; in fact, the MPV is inversely proportional to the degree of PLT maturity. A decrease in MPV is seen in conditions which reduce PLT production in the bone marrow. Van der Lelie et al showed that MPV was elevated in 13 of the 25 septicemia patients, and returned to normal values as soon as the disease was under control [12]. Another study of 10 infected patients with thrombocytopenia, found that MPV rose at the beginning and subsequently decreased, following a biphasic change [17]. In 2 different newborn cohorts with sepsis, thrombocytopenia and high MPV appeared to be prominent features [15], [16]. MPV is not increased in local infection or sepsis with negative blood culture. An elevation of MPV therefore suggests that the infection is invasive, systemic and uncontrolled and is related to the severity of the disease, a finding which was verified in our study, and may be useful as an assessment tool for prognostic features of septic shock. As stated earlier, one study showed that MPV falls as platelet count also decreases [10]. Therefore, previous studies are inconclusive in regards to MPV and have shown any number of MPV change during sepsis (increase, decrease, biphasic).

The discrepancies found in the literature may be due to different laboratory methods used. Studies have shown that the normal range of MPV should be established and calibrated within each specific laboratory due to the different laboratory analysis techniques [6], [18]. MPV changes are complex, and are not only related to the PLT count, but also related to the method of laboratory analysis used [19], [20].In a study by Akarsu et al, a MPV >9.5 fl was considered above normal range [15], and in another study, MPV elevation was defined >10.4 fl [7], both of which were commonly found in our study. The normal range of MPV from our laboratory was 9–17 fl, a range which is unsuitable for monitoring the evolution of septic shock. Most patients from our cohort had a MPV within the normal accepted range. Nevertheless, despite the absence of critical abnormalities in the MPV, discreet changes in MPV conferred prognostic value, as previous studies have suggested [12], [15], [16].

Platelet distribution width (PDW) is an indicator of the heterogeneity in platelet size. A high value of PDW suggests a large range of platelet size due to swelling, destruction, and immaturity. In our study, PDW was more elevated in non-survivors. Our finding is similar with that of Akarsu's research in neonates with sepsis [15].

Platelet large cell ratio, PLCR, is often correlated to MPV but is more sensitive to changes in platelet size. Babu et al has shown its level is inversely related to the platelet counts and directly related to MPV and PDW, and is an aid for the differentiation of thrombocytopenia [22]. This value was also more elevated in the non-survivor group. However the evidence concerning changes to PLCR as compared with MPV and PDW in septic states is limited. So further investigation is needed. The plateletcrit, PCT, is nonlinearly correlated to the platelet count and has a similar clinical implication. The PCT was also decreased in the patients who expired.

Among the most important findings of our research was a comparison of the five major platelet indices. The MPV had the highest precision rate of 75.6%, and the highest AUC (0.81) followed by PLCR (0.76) and PDW (0.75). As a predictive prognostic measure, a MPV cutoff was suggested to be over 10.5, over which there may be reasonable expectation of mortality. Though the AUC of PDW and PLCR are lower than MPV, they also may prove to be useful assessment tools in patients with sepsis. Previously Aydemir et al studied only the kinetics of platelets and MPV [7], whereas our study analyzes all five platelet indices.

The prognostic power of MPV was also compared to other usual indices of the complete blood count in septic shock. We found that the power of WBC count and HCT were lower than that of MPV. A possible explanation could be that white cell count may be affected by factors other than infection, such as stress and corticotherapy. HCT is affected not only by the hemoglobin level, but also by blood volume [23].

Since MPV showed the biggest power of all the PLT indices, it was compared with lactate, APACHE II score and procalcitonin. Although the APACHE II score has been used in prediction of sepsis outcomes, our study show that MPV is more precise. Giamarellos-Bourboulis et al have suggested that the APACHE II score combined with parameters such as serum soluble urokinase plasminogen activator receptor demonstrated higher accuracy for prognosis than APACHE II score alone [24]. The perceived uncertainness of the APACHE II score is the affection from the medical behaviors, especially for the patients transferred from other hospital [25]. The APACHE II score in our study was based on retrospective data that is available within 24 hours of ICU admission, which may affect the accuracy of APACHE II. Procalcitonin is a marker of infection severity, and has been used to predict the prognosis in septic shock. In our study, its ROC power of procalcitonin was lower than of MPV [26]. Lactate and its clearance had been used as important indices and added into the septic shock treatment guideline [1]. We found that lactate has more prognostic power than that of MPV (AUC of 0.81 for MPV versus AUC of 0.91 for lactate). MPV ranks the second after lactate which implies that it can be used in as a potential index. MPV is expected to constitute a good and simple index in combination with lactate.

### Limitations of our study and future work

Patients who received red blood cells and platelet transfusion were excluded as these directly affect the concentration of blood indices. Because this group may have more severe features, they should be investigated before a final conclusion is drawn about platelet indices changes in septic shock. Some therapeutic drugs including antibiotics and other baseline parameters like gender and age may have an impact on the platelet indices and could have affected our results [20]. A prospective study with a larger sample of adult septic shock patient, which includes criteria such as the infection sites, various therapeutic agents, and causative pathogens, is needed to compare their impact on severity.

## Conclusions

In this retrospective study, the rise in MPV, and to a lesser extent an increase in PLCR and PDW, is indicative of a worse prognosis in patients with septic shock. A statistical difference in MPV was seen between the non-survivors and the survivors of septic shock. After comparison with traditional prognostic markers of sepsis, MPV was found to be more closely correlated with mortality, only second to lactate. This suggests that the MPV may be a useful additional assessment tool and prognostic indicator of the outcome of septic shock patients.

## “What this paper adds” box

1: What is already known on this subject. Platelet can be decreased in septic shock, but there are few studies exploring the temporal changes of all various platelet indices and their results were contradictory.

2: What this study adds. Platelet indices changed more in the non-survivor group than in the survivor group, and a statistical difference of MPV was observed between the two groups. The MPV was shown to be strongly correlated to mortality, and can be used as a prognostic indicator.
