# Correction: The Impact of Various Platelet Indices as Prognostic Markers of Septic Shock

**DOI:** 10.1371/journal.pone.0117889

**Published:** 2015-02-03

**Authors:** The PLOS ONE Staff

The image for Fig. 3, “MPV, lactate, HCT, WBC, APACHE II and procalcitonin ROC curve map,” is incorrect. Please see the corrected Fig. 3 here.

**Fig 3:**
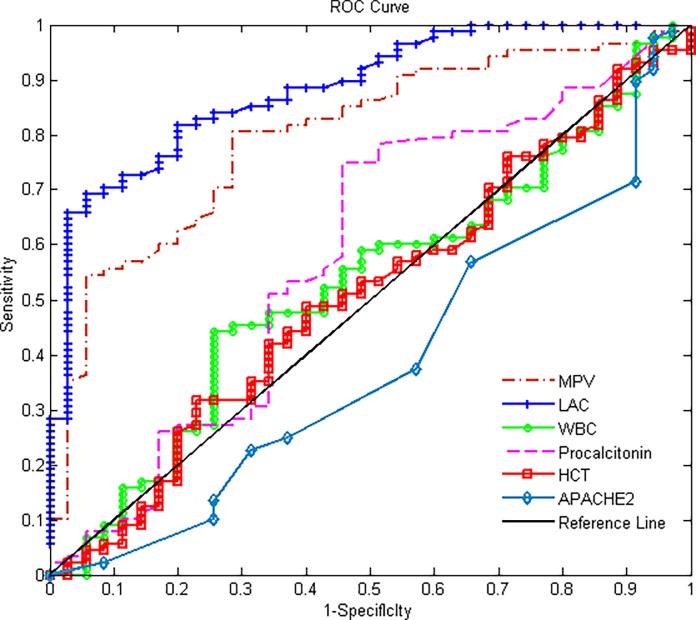
MPV, lactate, HCT, WBC, APACHE II and procalcitonin ROC curve map.

The following information is missing from the Funding section: This study was supported by the National Key Department Foundation of Health Ministry (No.2012-650,649).
